# Selective stabilization of aliphatic organic carbon by iron oxide

**DOI:** 10.1038/srep11214

**Published:** 2015-06-10

**Authors:** Dinesh Adhikari, Yu Yang

**Affiliations:** 1 Department of Civil and Environmental Engineering, University of Nevada, Reno, 89557, USA

## Abstract

Stabilization of organic matter in soil is important for natural ecosystem to sequestrate carbon and mitigate greenhouse gas emission. It is largely unknown what factors govern the preservation of organic carbon in soil, casting shadow on predicting the response of soil to climate change. Iron oxide was suggested as an important mineral preserving soil organic carbon. However, ferric minerals are subject to reduction, potentially releasing iron and decreasing the stability of iron-bound organic carbon. Information about the stability of iron-bound organic carbon in the redox reaction is limited. Herein, we investigated the sorptive interactions of organic matter with hematite and reductive release of hematite-bound organic matter. Impacts of organic matter composition and conformation on its sorption by hematite and release during the reduction reaction were analyzed. We found that hematite-bound aliphatic carbon was more resistant to reduction release, although hematite preferred to sorb more aromatic carbon. Resistance to reductive release represents a new mechanism that aliphatic soil organic matter was stabilized by association with iron oxide. Selective stabilization of aliphatic over aromatic carbon can greatly contribute to the widely observed accumulation of aliphatic carbon in soil, which cannot be explained by sorptive interactions between minerals and organic matter.

Soil organic matter (SOM) constitutes a major part of total carbon in the terrestrial ecosystem and the dynamics of SOM are crucial to global carbon cycle. SOM reserves 1500 Pg (1 Pg = 10 ^15^ g) carbon, more than the total amount in terrestrial vegetation and atmospheric environment^1^,^2^,^3^. The potential capacity of soil to sequester anthropogenic carbon can achieve as much as 55–78 Pg, given the total anthropogenic emission of carbon is around 244 Pg during the period of 1800–1994^4^. Decomposition of SOM releases 3.5 Pg/year carbon as CO_2_, a significant input of greenhouse gas to the atmosphere^2^. Soil-air carbon flux could be elevated significantly in the future with the predicted temperature increase, as the warming processes may accelerate the microbial decomposition and alter the chemical composition of SOM^5^,^6^. Therefore, better knowledge on the fate of SOM is key for accurately modeling and predicting carbon cycles under global climate change^4^.

A major challenge for modeling the SOM fate originates from a large uncertainty in understanding about the stability of SOM, with the residence time of SOM ranging from less than a year to hundreds of years^7^,^8^. It is unclear what factors control the stability of SOM in natural environments, although many hypotheses have been postulated regarding the stabilization of SOM^9^,^10^,^11^. Mounting evidence supports that the stability of SOM is an ecosystem-level property, which is mainly determined by ways that organic matter interacts with surrounding environment, especially minerals in soil^8^. The biogeochemical processes of organic carbon are closely coupling with the reactions for inorganic metals, which play an important role in stabilizing and destabilizing SOM^8^,^12^,^13^. To fully understand the mechanisms that minerals stabilize SOM, can help reduce the uncertainty in predicting the turnover of SOM and carbon cycling.

Iron oxide minerals have been suggested as an important regulator for the stabilization of SOM^14^. Correlation between the sorption of organic carbon and content of iron oxide minerals indicates the importance of iron oxides in accumulation and stabilization of organic carbon in soils^9^. Higher sorption capacity of iron oxides compared to other minerals enhanced its importance in the biogeochemical cycles and stability of SOM^15^. It was found that over 20% of organic matter in soil and sediment was stored by interactions with reactive iron minerals^9^,^16^. At the same time, iron oxide can be reduced by various microorganisms that gain energy by reducing iron^17^,^18^. Although the redox reactions can potentially break down the organic matter-iron oxide complexes and compromise the stability of SOM, there is limited information about how the reduction of iron oxide affects the stability of iron-bound organic matter.

Herein, the main objective of this study is to investigate the stability of iron-bound organic matter during the redox reactions. We found that aromatic carbon was selectively released during the reduction of hematite, although hematite preferred to sorb aromatic organic matter, because of stronger sorptive interactions.

## Results and Discussions

We evaluated the association with hematite and stability of three different humic acids, namely HA1-3, previously extracted from a peat soil and extensively characterized^19^,^20^. From HA1 to HA2 to HA3, we found an increasing trend in the fraction of aliphatic carbon content and a decreasing trend for aromatic carbon (Supplementary Table S1). HA1 and HA2 had relatively higher sorption on hematite compared to HA3 (Supplementary Fig. S1), indicating the importance of aromatic carbon in association between HAs and iron oxide. Sorption of HAs on hematite led to chemical fractionation of HAs, as a result of interactions between different functional groups of HAs and iron oxide. For all samples, values of *SUVA*_254_ -an indicator for the aromatic fraction of aqueous organic matter- decreased substantially after sorption on iron oxide (Supplementary Fig. S2, S3). With 100 mg C/L original aqueous organic matter, the sorption decreased *SUVA*_254_ from 5.04 to 0.76, 5.49 to 4.14, and 5.55 to 0.85 for HA1 at pH 5, 7, and 9, respectively (Supplementary Fig. S2). This decrease in *SUVA*_254_ corresponded to the change in the fraction of aromatic carbon from 36% to 8–31% based on the reported regression between the fraction of aromatic carbon and *SUVA*_254_^21^. Low values for relative *SUVA*_254_ (*SUVA*_254 R_), the ratio of *SUVA*_254_ for samples after sorption to those for original, quantitatively reflect the sorption-led decrease in aromatic fractions of HAs (Supplementary Fig. S3). Much smaller aromatic carbon fraction of HAs after the sorption on hematite was caused by the preferential sorption of aromatic organic matter on iron oxide compared to other fractions of organic matter. Such selective sorption, consistent with sorption isotherm analysis, supports the importance of aromatic carbon in the association between HAs and hematite.

**Figure 1:**
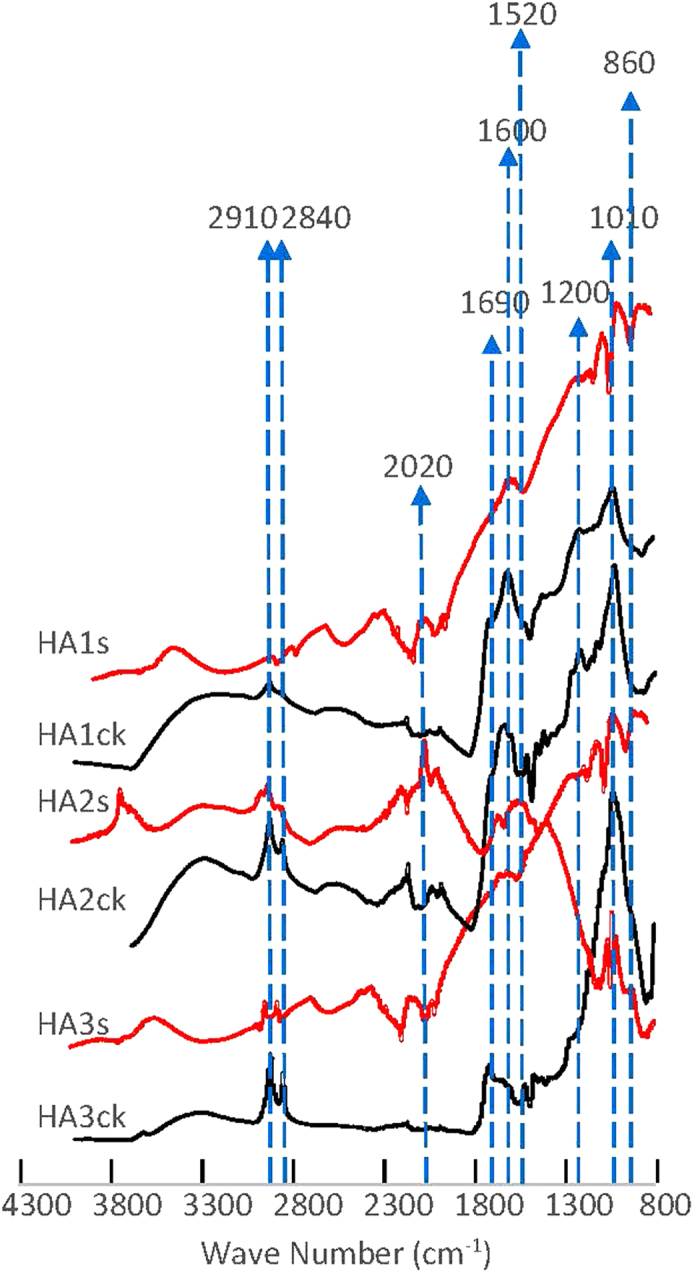
Attenuated total reflectance-Fourier transform infrared spectroscopy (ATR-FTIR) for original organic matter (HA1ck, HA2ck and HA3ck) and hematite-bound samples (HA1s, HA2s and HA3s) with the highest organic carbon content.

We used attenuated total reflectance-Fourier transform infrared spectroscopy (ATR-FTIR) to characterize the chemical structure of original and hematite-bound HAs (Fig. 1). For hematite-bound HAs, the peaks for alkyl carbon at 2910 and 2840 cm^−1^ were relatively weak compared to the original samples, indicating that the alkyl fractions decreased upon sorption^22^. Similar shape and symmetry intensity of the peaks at 2910 and 2840 cm^−1^ for original and hematite-sorbed HAs are recognized as a result of non-specific interactions between aliphatic carbon and iron oxide. The intensity of peak at 1600 cm^−1^, corresponding to the stretching of C=C in aromatic rings, decreased from HA1 to HA2 and HA3—consistent with the NMR-based fractions of aromatic carbon (Supplementary Table S1). For hematite-sorbed HA1 and HA3, the peaks at 1600 cm^−1^ were still prominent or even enhanced compared to the original HA samples. Such comparison suggests the relative enrichment of aromatic carbon upon sorption by hematite, together with the sorption isotherm and *SUVA*_254_ analysis. The peak for aromatic carbon shifted from 1600 to 1520 cm^−1^ for HA2 as a response to the sorption by iron oxide, which may be caused by the inner-sphere coordination complexation between phenolic carbon and iron. Peak splits at 1010 cm^−1^ for hematite-sorbed HAs compared to original HAs indicate the inner-sphere coordination complexation between hydroxide carbon and iron^23^. Consistently with the results of sorption isotherm, *SUVA*_254_ analysis and differences between ATR-FTIR spectra for hematite-sorbed HAs and original HAs prior to the sorption confirmed that aromatic fractions were preferentially sorbed by hematite. Such selective sorption can be a result of coordination complexation between phenolic carbon and iron as well as electron donor-receptor interactions between aromatic rings and iron oxide^24^. Aromatic carbon in soil is mainly originated from plant-derived lignin and black carbon produced by fires, while alkyl and carboxylic carbon is contributed by plant wax and microbial cell walls^25^,^26^. Our results showed that iron oxide preferred to bind with lignin- or black carbon-originated aromatic moieties.

**Figure 2:**
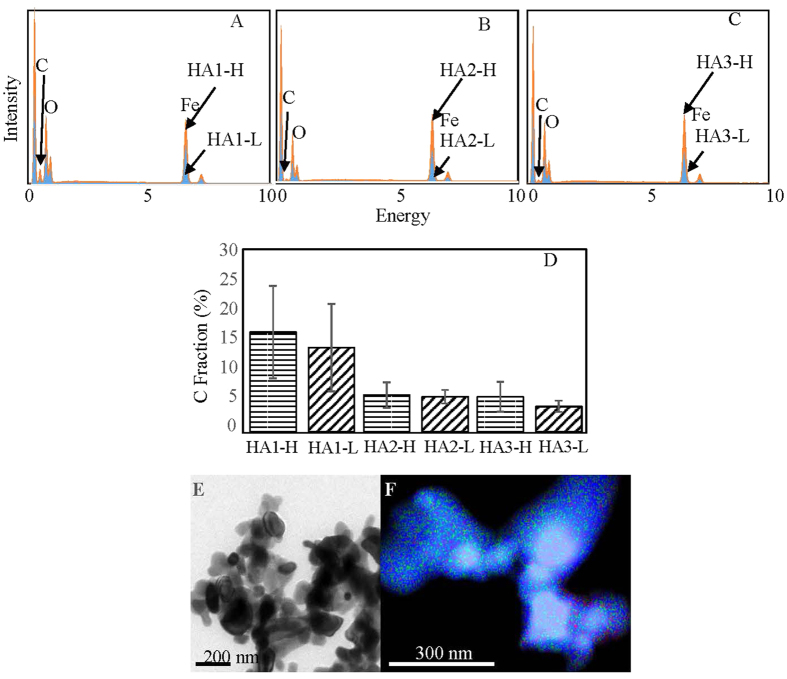
(**A**–**C**) Surface chemical compositional analysis for hematite-organic matter complex, determined by scanning electron microscopy coupled with energy dispersive spectroscopy (SEM-EDS). (**D**) Comparison of surface carbon composition for samples with highest and lowest bulky carbon content, based on EDS analysis. HA1-H, HA2-H, and HA3-H represent samples with highest carbon content, and HA1-L, HA2-L, and HA3-L stand for those with lowest carbon content. (**E**–**F**) Transmission electron microscope (TEM) observations for particles of HA3-hematite complex (**E**) and the EDS-based elemental distribution (**F**), with blue for iron, green for oxygen, and red for carbon.

Based on energy dispersive spectroscopy (EDS) analysis, iron contributed 36–69% of atoms on the surface of hematite-organic matter complex (Fig. 2A–D). When we compared the samples with the lowest and highest loading of organic carbon, the surface carbon fraction changed slightly–although the bulky carbon content increased by around 30 fold. Such discrepancy indicates that organic matter was not sorbed in single-layer mode, because single-layer sorption of organic matter would lead to covariance of surface with bulky carbon content. It is likely that organic matter formed multi-layer coating on the surface of hematite. Organic matter was sorbed directly by iron oxide and also on iron-bound organic matter. This was supported by the heterogeneous distribution of carbon on hematite, observed by a transmission electron microscope (TEM) (Fig. 2E,F). As shown in Fig. 2F, carbon was concentrated in the edge area of hematite particles, rather than homogeneously distributed on whole complexes.

We used abiotic reduction reaction with a relatively low ratio of sodium dithionite to hematite (1:0.8) to analyze the resistance of iron-bound organic carbon to the reduction process^16^,^27^. Under such experimental condition, only 15% of iron was reduced and released for pure hematite particles within a 24-h reaction period. For hematite-HA complexes, ~31–50% of iron oxide was reduced. The presence of organic matter facilitated the reduction-release of iron, likely through electron-shuttling effects^28^,^29^,^30^. Simultaneously, iron reduction caused 5–44% of carbon to release to the aqueous phase. The resistance of hematite-bound HA can be governed by its chemical composition and consequent molecular-level associations between iron and carbon.

**Figure 3:**
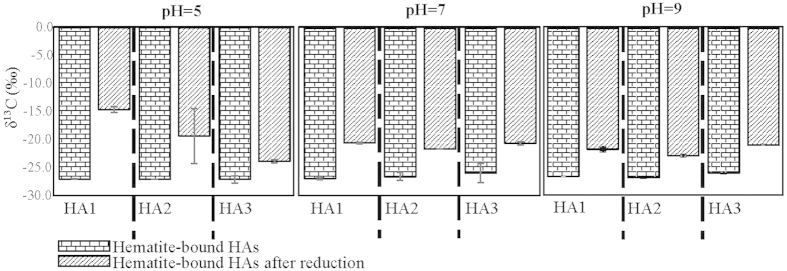
δ^13^C for hematite-bound organic carbon and the residual fractions after reduction-release experiments.

There is large difference in the stabilities of different functional groups of iron-bound SOM during the reduction reaction. Based on the δ^13^C analysis, the reduction-resistant fractions of iron-bound HAs were relatively enriched in ^13^C compared to the original HAs (Fig. 3). At pH 5, δ^13^C for residual iron-bound HA1–HA3 was −14.56,−19.31, and−23.78‰, while the original values were −26.26, −25.30, and −25.03‰ for HA1, HA2, and HA3, respectively. Previous studies showed a strong negative correlation between the value of δ^13^C and old organic matter with higher lignin content, which is a major contributor for aromatic moieties in soil/sediment organic matter^31^,^32^. In this study, we developed an index for the amount of aromatic carbon in original hematite-bound organic matter:
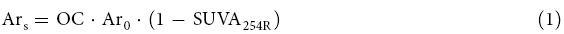
where *Ar*_S_ is the index for the amount of aromatic carbon in hematite-HA complexes, *OC* is the organic carbon content bound by iron oxide obtained by the sorption isotherm analysis, and *Ar*_0_ is the original aromatic carbon fraction in HAs derived from our previous NMR analysis^19^ (Supplementary Table S1). As we discussed, *SUVA*_254R_ represents the relative decrease in aromatic fraction of aqueous HAs after sorption, and therefore (1 −*SUVA*_254R_) is used to represent the degree, by which the aromatic carbon was enriched in iron-bound HAs. We found a negative correlation between *Ar*_S_ and δ^13^C for iron-bound organic matter before reduction (Pearson Correlation Coefficient = −0.41, *p* < 0.05, Supplementary Fig. S4), which further demonstrated the applicability of δ^13^C as an index for aromatic fractions in residual organic matter. Therefore, the increase in δ^13^C of HAs after the reduction reaction indicates that residual organic matter had less aromatic carbon compared to the original iron-bound HAs. These results suggest that the aromatic carbon-rich organic matter was relatively easily released in the reduction reaction, although hematite selectively sorbed a larger amount of aromatic organic matter compared to other components of HAs. This finding implies contrast stability and sorption of aliphatic and aromatic organic matter on hematite. Resistance to reduction release represents an un-presented mechanism that aliphatic carbon was reserved and stabilized in soil environment. Aromatic fractions were selectively released by the reduction reaction, although they were sorbed more preferentially on hematite. The susceptibility of aromatic organic matter to reduction may be a result of the steric effect of aromatic rings, which lead them to mainly locate on the outer surface of the organic matter-mineral complex, while the more flexible aliphatic components can reside in the inner-layer immediately bound to iron oxide and be preserved by outer-layer organic matter. Such multi-layer scheme is supported by our SEM/TEM-EDS analysis.

Here we presented contrasting sorption and stabilization of aliphatic and aromatic organic matter on hematite. We discovered that hematite preferred to sorb more aromatic organic matter as a result of inner-sphere coordination and other interactions, but the aromatic carbon-rich organic matter was more susceptible to the reduction release. These results have important implications for the biogeochemical cycle and stabilization of carbon. First, we provided evidence that iron-bound, non-aromatic carbon was more resistant to reduction reactions, which can preserve aliphatic organic matter. This finding partially explains the widely observed accumulation of aliphatic organic matter in soil, unexplained by the sorptive interactions between aliphatic organic matter and mineral phases^33^,^34^. Second, the discrepancy in sorption and stability in redox reactions warns that sorption cannot be used independently as an indicator or as a predictor of the stability of carbon. Third, we suggested a paradigm for the organic matter-mineral complex – with the flexible aliphatic fraction residing in inner-sphere layer and rigid aromatic carbon on the outer surface. Such scheme for the interactions between organic matter and iron oxide can be valuable for evaluating the biogeochemical fate of iron oxide-organic matter complexes.

## Methods

### Preparation of HA stock solution

Alkaline extraction method was used to prepare all stock solutions of HA used in this study. An aliquot of HA1 was mixed with 0.3 mL of 3 M sodium hydroxide solutions. The mixture was sonicated for 1 hour. After sonication, mixture diluted with 1 mL of DDI water was shaken at 100 rpm for 24 hours. Then, the solution was centrifuged at 3000 rpm for 20 min to obtain the particle-free supernatant. Organic carbon concentration in stock solutions were measured by Shimadzu TOC-VCSH (Kyoto, KYT, Japan). The solution was stored at 4°C prior to the experiment.

### Sorption experiment

Sorption isotherm for three HA samples on hematite particles were obtained at pH = 5, 7 and 9. Pre-determined volume (less than 1 mL) of HA stock solution was mixed with buffer solution of 3.7 mM CaCl_2_ and 1.5 mM NaN_3_ to make the final volume of 7.5 mL. Solution pH was adjusted using 1 M HCl and 1 M NaOH. To achieve suspending 0.03 g hematite/mL, the solution was mixed with 7.5 mL of 6% (w/v) hematite suspension with same pH values. The hematite suspension was made by suspending hematite particles in buffer of 3.7 mM CaCl_2_ and 1.5 mM NaN_3_ and stirring the suspension for at least 1 hour. Starting organic carbon concentration ranged from 10 to 100 mg/L. To make control samples, same procedures were followed as for samples, just without suspending hematite particles. The mixtures were shaken at 100 rpm at room temperature for 24 hours, which has been determined to be long enough to establish the sorption equilibrium of organic matter on hematite. After that, the samples were centrifuged at 10,000 rpm for 20 min and residual organic carbon concentration in supernatants were measured by Shimadzu TOC-VSCH. Residual hematite-HA complexes were collected and stored at 4 °C for further characterization and analysis. All the experiments were performed in duplicates. The sorbed amount of organic carbon was calculated as the difference between the organic carbon concentration in suspension before and after the sorption. For partial samples, the hematite-sorbed organic carbon content was also directly measured with an Eurovector elemental analyzer interfaced to a Micromass Iso-Prime stable isotope ratio mass spectrometer (EA-SIRMS) (EuroVector SpA, Via Tortona, Milan), which can transform all carbon to CO_2_ by combustion. Close relationship between the direct measurement for iron-bound carbon content and calculated sorption confirmed the sorption isotherm we observed (Supplementary Fig. S5). The sorption isotherm was fitted by a linear model:
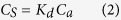
where *C*_s_ is the sorbed organic carbon content (mg/kg), *C*_a_ is the aqueous organic carbon concentration (mg/L) and *K*_*d*_ is the sorption coefficient (L/kg). The *K* was derived as an indicator for the sorption of organic carbon on hematite.

### Reduction release experiment

To examine the stability of hematite-sorbed organic matter, the reduction-release of iron-associated carbon was analyzed for hematite-HA complex with highest carbon content, following previously reported method^16^. Hematite-HA complex samples were dried for 40 hours at 49 °C. An aliquot (~0.2 g dry weight) of dried hematite was mixed with 15 mL buffer (pH = 7) containing 0.11 M NaHCO_3_ and 0.270 M Na_3_C_6_H_5_O_7_. Then, 0.25 g of sodium dithionite was added to the solution and the whole mixture was incubated in a water bath at 80 °C for 15 min. The ratio of sodium dithionite to iron was relatively low and the relatively stable fraction of hematite-HA will be resisting to the reductive reaction. After the reaction, the samples were centrifuged at 10,000 rpm for 10 minutes and supernatants were collected. The residues were washed three times using 5 mL DDI water and centrifuged at 10,000 rpm for 10 minutes. Supernatants were combined together. Washed residues were dried, weighed and measured for carbon content and δ^13^C using an EA-SIRMS. For the control, we used 0.2 g pure hematite to go through the same procedure for samples. Total mass of carbon before and after reduction release was calculated to get the released fraction of carbon. The mass of particle was used to quantify the released fraction of iron. δ^13^C was obtained as an indicator for the chemical nature of resisting carbon. The sorption of citrate on hematite was measured under the same condition just without adding dithionite and was used to calibrate the releasing fraction of carbon. δ^13^C of citrate used in this experiment was −24.9‰, below the values for most of samples after reduction, which validated our discussion for change in δ^13^C upon reductive release without calibration.

### ATR-FTIR

ATR-FTIR spectra were obtained for original HA samples and hematite-bound organic matter by a Thermo Scientific Nicolet 6700 FTIR (Waltham, MA). Spectra were acquired at the resolution of 4 cm^−1^ and resulted from 100 scans. Data collection and spectral calculations were accomplished using OMNIC software version 8.3.103.

## Additional Information

**How to cite this article**: Adhikari, D. and Yang, Y. Selective stabilization of aliphatic organic carbon by iron oxide. *Sci. Rep.* **5**, 11214; doi: 10.1038/srep11214 (2015).

## Supplementary Material

Supplementary Information
