# Supplementary material for: Selective stabilization of aliphatic organic carbon by iron oxide
Source: Sci Rep. 2015 Jun 10;5:11214. doi: 10.1038/srep11214 (PMC4462107; doi:10.1038/srep11214)
Supplement: Supplementary Information [file srep11214-s1.pdf]

SUPPLEMENTARY INFORMATION FOR

Submission to *Scientific Reports*

Selective stabilization of aliphatic organic carbon by iron oxide

Dinesh Adhikari[1], Yu Yang[1]★

1 Department of Civil and Environmental Engineering, University of Nevada, Reno, 89557, USA

★Corresponding Author: yuy@unr.edu

NUMBER OF FIGURES: 6

NUMBER OF TABLES: 1

NUMBER OF PAGES: 11

Materials

Hematite (α-Fe_2O_3) was purchased from Fisher Chemical (Fisher Scientific, Fail Lawn, NJ) and used as received. The mineralogy was confirmed by X-ray diffraction (XRD) analysis, obtained at a Bruker D8 Advance X-ray diffractometer (AXS Inc, Madison, WI) (Supplementary Fig. S6). The surface area was 1.9 m^2/g, determined by an AutoChem II 2920 (Micromeritics, Norcross, GA 30093-2901, U.S.A.). Humic acid (HA) samples, previously extracted from a peat soil, were used in this study, as model SOM. The extraction and characterizations for HA can be found in our previous publications[1,2]. Briefly, we extracted HAs from a peat soil, collected at Lawrence swamp in Amherst, Massachusetts. The peat soil was extracted 13 times with 0.1 M $Na_4P_2O_7$ and 0.1 M NaOH. Extracted HAs were precipitated at low pH and de-ashed with HCl/HF. In this study, we used the first, eighth and last extraction as HA1, HA2 and HA3. All these HAs have been characterized extensively by various techniques, including but not limited to elemental analysis, nuclear magnetic resonance (NMR) spectroscopy, X-ray photoelectron spectroscopy (XPS), and synchrotron-based near-edge X-ray absorption fine structure (NEXAFS). The physicochemical properties of HAs were summarized in Table S1 (Supporting Information (SI)). All the reagents, used in this study were analytical grade and purchased from Sigma-Aldrich (St. Louis, MO, US). Distilled deionized (DDI) water was used.

UV analysis

UV absorbance (190 nm to 600 nm) was analyzed for original HA solution (20 mg/L) and HA solution after sorption by hematite using a Thermo Scientific-Evolution 260 bio UV scanner (Thermo Electron Scientific Instruments, Madison, WI). A 1cm×1cm quartz glass cuvette was used for the analysis. The UV absorbance of buffer used in the sorption experiment was analyzed as background. Each sample was scanned twice. $SUVA_{254}$ (L/mg/m) was calculated as the following equation:

$$SUVA_{254} = \frac{A_{254}}{OC \cdot L} \quad (1)$$

where A_{254} is the absorbance at 254 nm, OC is the organic carbon concentration (mg/L), L is the depth of the cuvette (m). The $SUVA_{254}$ has been shown to be closely related to the aromatic carbon fraction determined by NMR. We also calculated $SUVA_{254R}$:

$$SUVA_{254R} = \frac{SUVA_{254s}}{SUVA_{254o}} \quad (2)$$

where $SUVA_{254s}$ is the $SUVA_{254}$ for the solution after the sorption, and $SUVA_{254o}$ is the value for original solution. The value of $SUVA_{254R}$ was calculated as an indicator for the residual fraction of aromatic carbon upon the sorption by hematite to represent the sorptive chemical fractionation.

SEM-TEM EDS analysis

SEM/TEM observation and EDS spectra analysis were applied to study the morphology and surface chemical composition of hematite-organic matter complex. The samples were directly loaded to the silicon chip for SEM/TEM-EDS analysis. SEM-EDS analysis was obtained by using a Hitachi S-4700 II (Hitachi Ltd. Tokyo, Japan) with an Oxford INCA energy-dispersive spectrometer. Electron beam energy was set at 12 KeV for analysis to mainly analyze carbon, oxygen and iron. The spectra were collected for 90 seconds. TEM observation was performed on a JEOL-JEM 2100F analytical TEM (JEOL, Peabody, MA, USA).

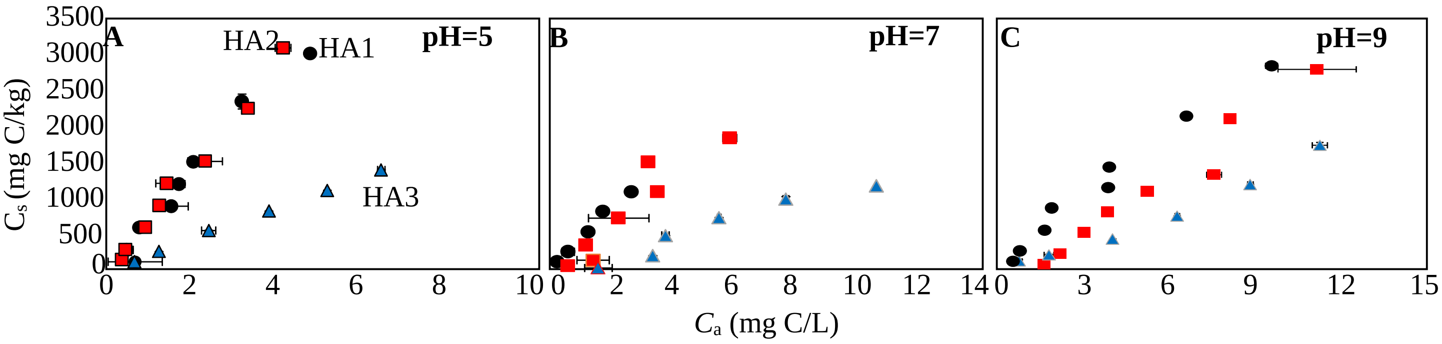

Fig. S1 Sorption isotherms of HA1, HA2 and HA3 on hematite under pH 5-9. C_a and C_s are equilibrium aqueous organic carbon concentration (mg C/L) and iron-bound carbon content (mg C/kg hematite). The bars represent the standard deviations obtained from duplicate experiments. Some of them are relatively small and hard to see compared to data spots in the figure.

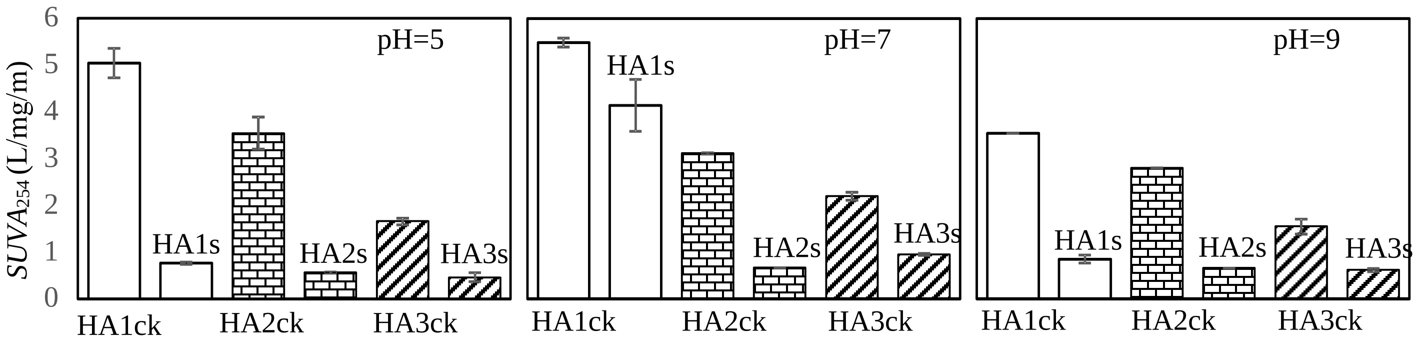

Fig. S2 Specific UV absorbance at 254 nm ($SUVA_{254}$) for original HA solutions and residual solutions after the sorption experiment, for the solution samples with highest organic carbon concentration. HA1ck, HA2ck and HA3ck represent original HA solutions. HA1s, HA2s, and HA3s represent HA solution after the sorption experiment.

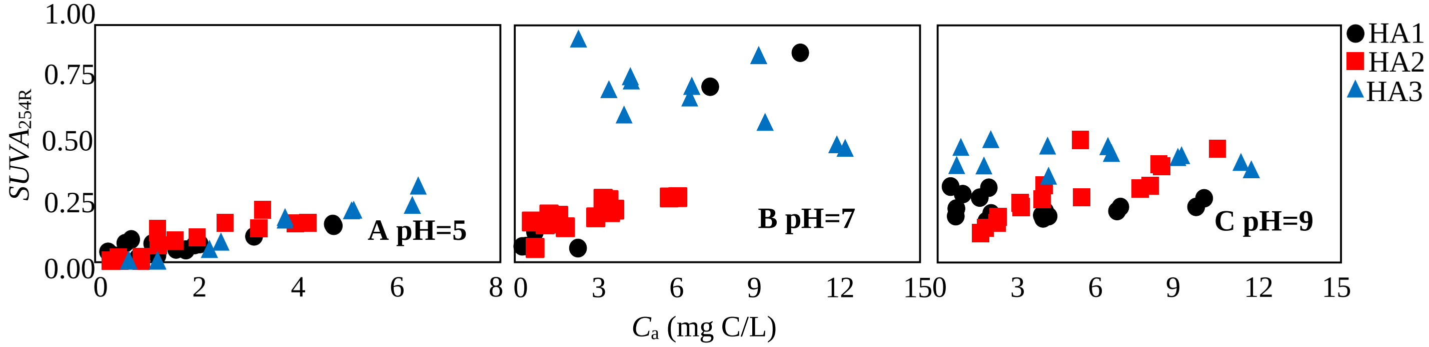

Fig. S3 Relative specific UV absorbance at 254 nm ($SUVA_{254R}$) as the ratio of $SUVA_{254}$ for samples after sorption to that for original samples. C_a is the equilibrium organic carbon concentration in solutions after sorption.

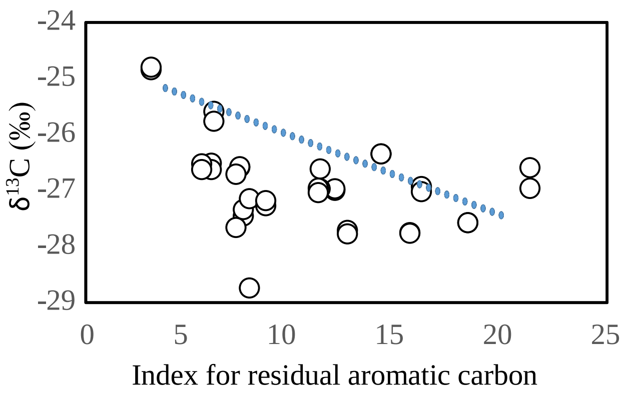

Fig. S4 Relationship between the $\delta^{13}C$ of iron-bound carbon and Ar_s (index for residual aromatic carbon fraction). Dashed line represents the regression.

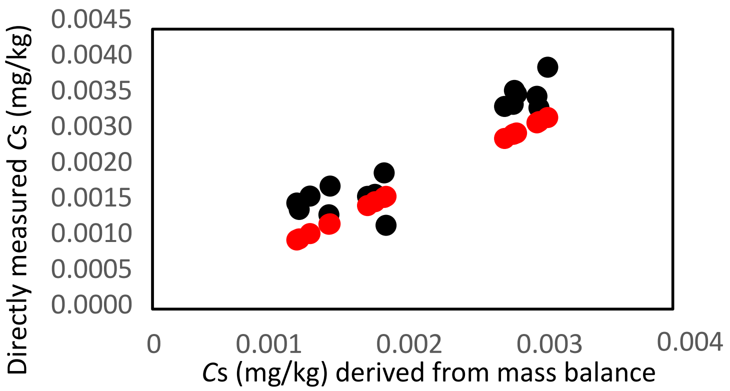

Fig. S5 Correlation between the directly measured carbon content by EA-SIRMS and the value obtained in the sorption isotherm analysis through mass balance. Black dots stand for samples, while red dots represent 1:1 ratio.

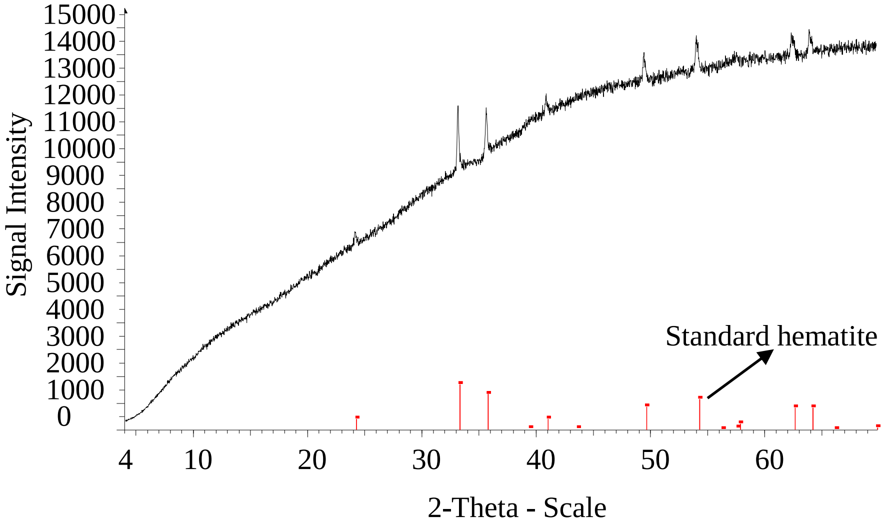

Fig. S6 XRD analysis for the iron oxide particles we have. It matched well with the spectra for standard hematite (shown as red sticks in the figure).

Table S1 Chemical composition of HA samples including elemental fraction and the fractions of each functional group detected by NMR analysis

	Elemental Analysis (%)				NMR-derived Fractions of Functional Groups (%)					
	C	H	N	O	Alkyl	O-alkyl	Aromatic	Phenolic	Carboxyl	Carbonyl
HA1	51.9	4.7	3.1	40.4	32	31	19	6	11	1
HA2	54.5	6.1	3.2	36.2	39	32	14	5	8	1
HA3	58.4	7.2	1.9	32.5	55	27	10	3	4	0
